# Psychological distress reported by healthcare workers in Saudi Arabia during the COVID-19 pandemic: A cross-sectional study

**DOI:** 10.1371/journal.pone.0268976

**Published:** 2022-06-03

**Authors:** Yasmin Altwaijri, Lisa Bilal, Amani Almeharish, Abdulrahman BinMuammar, Edward DeVol, Sanaa Hyder, Mohammad Talal Naseem, Areej Alfattani, Abdussalam Ali AlShehri, Rami Almatrafi

**Affiliations:** 1 King Salman Center for Disability Research, Riyadh, Saudi Arabia; 2 Biostatistics, Epidemiology and Scientific Computing Department, King Faisal Specialist Hospital and Research Centre, Riyadh, Saudi Arabia; 3 SABIC Psychological Health Research & Applications Chair (SPHRAC), College of Medicine, King Saud University, Riyadh, Saudi Arabia; 4 Prince Mohammed Bin Abdulaziz Hospital, Riyadh, Saudi Arabia; 5 King Faisal Hospital, Mecca, Saudi Arabia; Oxford University Hospitals NHS Foundation Trust, UNITED KINGDOM

## Abstract

**Introduction:**

Few studies have considered the impact of COVID-19 on the mental health of healthcare workers (HCWs) in the Kingdom of Saudi Arabia (KSA). We estimated the prevalence and severity of psychological distress and characterized predisposing risk factors among HCWs in KSA during the COVID-19 pandemic.

**Methods:**

We conducted a cross-sectional online survey of 1,985 HCWs from 6 hospitals across the country designated with caring for COVID-19 patients between April 16 and June 21, 2020. Our data analysis was performed using logistic regressions. Ordered logistic regressions were also performed using forward stepwise model selection to explore the effects of risk factors on psychological distress.

**Results:**

The prevalence of psychological distress reported by HCWs in KSA was high, ranging from mild-moderate to severe in severity. Younger HCWs, women, those in contact with COVID-19 patients, and those who either had loved ones affected or who were themselves affected by COVID-19 were the most at-risk of psychological distress. Risk factors such as insomnia, loneliness, fear of transmission, and separation from loved ones most significantly predicted elevated levels of distress among HCWs.

**Conclusions:**

Increasing psychological distress was commonly reported by HCWs during the early months of COVID-19 pandemic in KSA. Public health policy makers and mental health professionals must give special attention to risk factors that predispose HCWs in KSA to psychological distress.

## Introduction

The likelihood of developing a psychological injury versus experiencing psychological growth is influenced by the support received before, during, and after a difficult life event [1]. The COVID-19 pandemic has been an extraordinary challenge for populations around the world. At the focal center of fighting this disease are healthcare workers (HCWs). Protecting them, therefore, must be a crucial component of public health measures aimed at addressing the outbreak [2].

Several reports have discussed the psychological impact of COVID-19 on frontline HCWs [3–5]. The emotional response of HCWs to an outbreak of a disease like COVID-19 is complicated, with potentially long-term mental health implications [2]. Manifestations of psychological distress among HCWs include elevated levels of stress, anxiety, depression [6], severe insomnia, obsessive-compulsive symptoms, somatization [7], post-traumatic stress disorder [8], vicarious traumatization [9], increased risk of developing other mental health problems [1] and in the most vulnerable cases, suicide [10].

Hospitals nationally and globally were struggling with the effects of the pandemic and mental health crisis, including the general population. Women have been found to report more severe symptoms of depression, anxiety and psychological distress than men [3]. Although nurses may be more prone to developing unfavorable mental health outcomes [2], the evidence is mixed, as non-medically trained HCWs may also be at a higher risk for psychological distress during the COVID-19 pandemic in comparison to medically trained personnel [5].

Studies attribute the emotional strain experienced by HCWs to various reasons such as intensified perception of experiencing personal danger, widespread media coverage, inadequate support [2], reluctance to work or contemplating resignation [11], rising numbers of acutely ill patients, anxiety about assuming unfamiliar clinical roles, expanding workloads caring for COVID-19 patients, caring for affected coworkers [3], fear of transmitting the virus to fellow HCWs [8], shortages of medical equipment [3] such as personal protective equipment, limited testing and treatment options for COVID-19, fear of infecting family members due to workplace exposure, the pressure of making emotional and ethical resource-allocation decisions, work-related burnout [12], stigmatization and ostracism for displaying physical symptoms suggestive of COVID-19 infection [13, 14], and limited access to mental health services [3].

It is also important to consider these findings with respect to methodological rigour and quality checks (potentially due to rapid publication of research). For instance, one study reported findings drawn from a relatively small sample size with a low response rate from HCWs, and using measures with single-item ratings [6]. Nevertheless, there is a critical need for healthcare organizations and researchers to prioritize the mental health needs of HCWs serving the community during the pandemic globally [3]. There may even be the risk of the COVID-19 outbreak leading to a ’second pandemic’ of mental health crises in health systems and communities [15]. The implications of this study are needed to develop specialized psychological interventions for HCWs [2, 16], improve relevant organizational and management policies, strengthen and prepare healthcare personnel to provide psychological support and tackle mental health challenges [1, 17], and establish prevention strategies such as screening for psychological distress, as well as providing psychoeducation and targeted support to those at most risk [12].

## Methods

### Study design, sampling and participants

This study was a cross-sectional, hospital-based survey conducted using REDCap electronic data capture tools [18, 19] hosted at King Faisal Specialist Hospital & Research Centre (KFSH&RC), Riyadh. A total of 18,567 healthcare workers from 6 major hospitals ‒ KFSH&RC in Riyadh, Jeddah and Medina; King Fahad National Centre for Children’s Cancer, Riyadh; Prince Mohammed Bin Abdulaziz Hospital, Riyadh; and King Faisal Hospital, Mecca ‒ were all invited to participate in the study by completing an online questionnaire between April 16 and June 21, 2020. This is a convenience sample from six hospitals that were designated to provide healthcare services to high-risk COVID-19 patients and which agreed to participate in the project, in line with similar international studies [20–22].

Following recommendations by Pierce et al. [23] to devise robust sampling strategies for mental health surveys during COVID-19, the study was designed to include a sufficient number of respondents to estimate the prevalence and severity of psychological distress among HCWs in the KSA during the pandemic. The goal of the study was to estimate the prevalence of psychological distress with a 99% confidence interval to within three percentage points, and to generate a proportional odds model for non-modifiable correlates predicting psychological distress with a 95% confidence interval. A conservative (with respect to required sample size) assumption of 0.50 for the prevalence was used in the calculation. Given this, the required sample size was 1,843 participants. This study was undertaken with the intention to collect this number of respondents.

Key events during the outbreak of COVID-19 in KSA and the study timeline are shown in the supplement (S1 Fig). The study and its procedures were approved by the Institutional Review Board at the KFSH&RC, Riyadh (RAC#: 2091093, April 12, 2020). Participants were assured that their data would be kept anonymous and confidential, and they provided written informed consent by marking the required checkbox prior to answering the survey questions.

### Study measures

Study measures were developed using face validity. A committee of experts and qualified authors with backgrounds in psychology, survey methodology, and epidemiology judged the instrument to be appropriate for the target objectives and assessment. While expert judgement should not always be used as a substitute for content validity, it can be used when research is conducted urgently and within a limited timeframe [24].

A self-administered questionnaire was developed, and the online survey link was circulated via institutional/hospital email networks through a preliminary email followed by two reminder emails. Upon clicking on the link, the study and its objectives were described. The duration to complete the survey (approximately 5 minutes), and the contact information for researchers were also provided. The designed questionnaire was enabled when participants chose to "agree" to participate in the survey, and no information was collected if the participant chose to "disagree" and not give consent to participate. The questionnaire consisted of the following:

### Demographics

Self-reported demographic data included gender, age, marital status, type of healthcare personnel (physician, nurse, allied health professional, researcher or other–specify), name of the hospital and department of employment (clinical or non-clinical), and work mode in the past 30 days (completely tele-working from home, completely working in the hospital, working from home and the hospital, or not working at all). Details of healthcare personnel under each category and distribution of sample according to department can be found in the supplement (S1 Table).

While we cannot evaluate which demographic characteristics are over- or under-represented, we do know that nurses and allied health professionals make up most of the sample our study is based on, and the distribution of our sample is in line with official country statistics [25].

### Study context

The Kingdom of Saudi Arabia (KSA) began undertaking nation-wide measures to curb the spread of COVID-19 in March, 2020, when the first case of infection was confirmed [26]. Strict curfews, restricting mobility and travel [27], temporary suspension of prayers in local mosques and pilgrimage to the holy cities of Mecca and Medina [28], and closure of educational institutions [29] were some of the major measures implemented by the KSA [30]. The curfew restrictions did, however, allow movement and travel for those working in specific sectors, including the health sector [31]. According to national data in the year 2020, physicians made up 20.6% of all HCWs, while dentists made up 4.2%, nurses (including midwives) made up 42.5%, pharmacists made up 5.9%, and allied health personnel made up 26.8% [25]. The country’s HCWs that could work remotely were requested to stay home and those found at-risk were asked to quarantine [30]. The aim of our study is to report the prevalence, severity and risk factors of psychological distress among HCWs in KSA during the COVID-19 pandemic.

### COVID-19 exposure

Questions related to exposure to COVID-19 asked: (i) whether or not the HCW had been directly engaged in the diagnosis, treatment, or care for COVID-19 patients, (ii) if the health of the HCW was affected by COVID-19 (Yes/No) and if so, to indicate if they were infected, hospitalized and/or quarantined by health authorities, and (iii) if the health of the HCW’s loved ones had been affected by COVID-19 such that a loved one was (a) infected, (b) hospitalized, (c) in quarantine due to exposure, (d) died or I the question was not applicable.

### Psychological distress

The K6, a psychometrically reliable and valid scale, which was used in the Saudi National Mental Health Survey questionnaire the CIDI 3.0 and validated for use in KSA [32, 33], was used to measure psychological distress; the scale is reported to be a well-known indicator correlated with the presence of a diagnosable mental illness [34–36]. Participants were asked to consider the past month and to report how frequently they had experienced the following symptoms: felt nervous, hopeless, restless or fidgety, worthless, depressed, and felt that everything was an effort [34]. For scoring the items, a value of 0, 1, 2, 3, or 4 was assigned to ’none’, ’a little’, ’some’, ’most’, or ’all’ of the time, respectively; responses to all six items yielded a K6 score between zero and 24, where the total score of 13 or more indicated a greater tendency towards mental illness [35, 37, 38], a table including K6 score means can be found in the supplement (S2 Table).

### Other correlates

The questionnaire also included an item related to insomnia asking the HCW to indicate (always/sometimes/never) if in the past 30 days they had difficulty falling asleep at night, waking up during the night, waking up too early, feeling sleepy during the day, or not feeling well-rested after a night’s sleep. Another item assessing their financial situation asked to indicate which of the following applied to them: they or another jobholder in the household (a) lost a job, were laid off, or had hours reduced, (b) had an increase in work hours, (c) experienced another loss of income (retirement payments, stocks, other investments), or (d) the statements were not applicable to them. Questions measuring other types of emotional strain were introduced by asking the participant to report how worried they were about the COVID-19 currently: very/somewhat/not very/not worried at all. Similarly, they also rated ’worries me a lot/somewhat/does not worry me at all’ for the following COVID-19-related concern items: they will get infected with COVID-19, they will infect the people close to them, unavailability of personal protective equipment (masks, gloves, gowns, and eye wear), family and friends will become infected with COVID-19, they or their relatives or friends will die from infection with COVID-19, feeling lonely and bored and missing being with friends, being far from family and the people they love because of the travel ban, losing their job and other financial resources, inability to obtain food and supplies needed for themselves or their family, inability to obtain medical care or medications for themselves or their family, continuation of the COVID-19 pandemic for a long time, decline in their fitness level and gaining weight due to self-isolation, not practicing favorite hobbies due to self-isolation, the world not returning to what it was before the COVID-19 pandemic, and being stigmatized if infected with COVID-19.

### Statistical analysis

Data analysis was performed using PROC FREQ and PROC LOGISTIC procedures in SAS version 9.4 (SAS Institute Inc., Cary, NC). Descriptive analysis was conducted to report frequencies and percentages, a table including the variable frequencies can be found in the supplement (S3 Table). Prevalence rates for psychological distress were reported as proportions, with standard errors and 99% confidence intervals. An ordered logistic regression or proportional odds model with the 3-level ordinal K6 score (ordered—coded as: Severe—1, Mild/Moderate—2, No distress—3) as the outcome was used to generate odds ratios with 95% confidence intervals for non-modifiable correlates (demographic and COVID-19 exposure–related variables). Ordered logistic regressions were also performed using forward stepwise model selection to explore the effects of risk factors on psychological distress. We ran the proportional odds model assuming a fixed odds ratio across the three levels of the K6 (no stress, mild/moderate stress, severe stress). That is, the ratio of the odds for mild/moderate stress (versus no stress) across levels of the factor of interest (as mentioned in Tables 3 and 4), is the same as the ratio of the odds for severe stress (versus mild/moderate stress) across the same levels of the factor of interest. A test of this assumption was carried out for each of the models and yielded no significance (p > 0.05) except for the model where one of the variables was about stigma (S4 Table). Since the stigma variable was not found to be significant in the assumption, we ran it as a binary logistic regression, and it was found to be significant. For this model, the K6 scale was dichotomized into Mild/Severe stress and No stress (S5 Table). The reduced model consisting of non-modifiable factors (gender, age, marital status, COVID-19 contact, healthcare personnel, hospital department, someone close affected by COVID-19, and self-affected by COVID-19) was first selected, and then modifiable factors (such as insomnia, financial situation, and work mode) were introduced en masse, as well as individually to explore incremental changes in effects. The likelihood ratio of each full model was subtracted from the likelihood ratio of the reduced model to estimate the effect of specific variable(s) on psychological distress. We categorized the severity of psychological distress based on the total score as no/low distress (score 0–4), mild/moderate distress (score 5–12) and severe distress (score 13–24).

## Results

**Fig 1:**
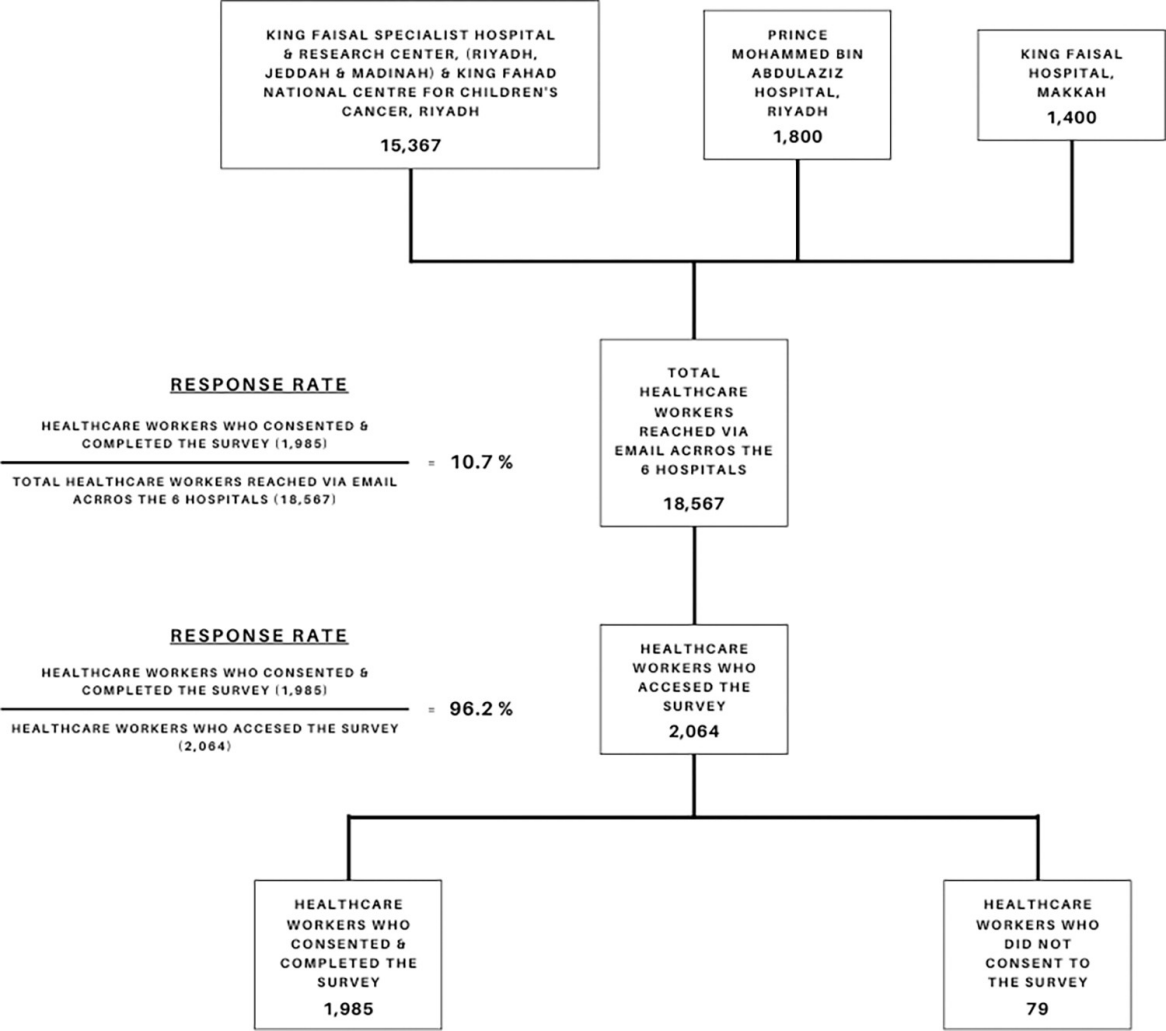
Response rate.

Seventy-nine individuals chose the ’disagree’ option at the beginning of the survey, not giving their consent to participate. Out of the 2,019 participants who did provide informed consent, 1,985 HCWs completed the entire survey; these were used to conduct data analyses Fig 1.

As shown in Table 1, the sample was predominantly female, aged 30–39 years old, and married. The mean age of the participants was 38.67±9.83 years old (further details on age means included in the supplement, S2 Table). With respect to their work mode in the past 30 days, half of the HCWs had been working within the hospital premises. Nurses and allied health professionals made up most of the sample. Roughly half of the HCWs belonged to clinical hospital departments; out of which (n = 1077), most worked in the Department of Medicine (consisting of subspecialty units for Allergy/Immunology, Endocrinology, Gastroenterology, Infectious Diseases, Internal Medicine, Nephrology, Pulmonary Medicine, and Rheumatology), Critical Care Medicine, Surgery, Pathology & Laboratory medicine, and Oncology (further details included in the supplement, S1 Table) Table 1. In terms of COVID-19 exposure, one-quarter of the HCWs had directly engaged in diagnosis, treatment, or care for COVID-19 patients. However, the majority of them had not contracted the infection themselves, and did not have close loved one(s) who had been affected by COVID-19 Table 1.

**Table 1:** Sample demographics and COVID-19 exposure.

Socio-demographic Variables	N	%
**Gender (N = 1978)**		
Male	756	38.2
Female	1222	61.8
**Frequency Missing = 7**		
**Age (N = 1927)**		
20–29	360	18.7
30–39	765	39.7
40–49	479	24.9
50–59	270	14.0
60–70	53	2.8
**Frequency Missing = 58**		
**Marital Status (N = 1980)**		
Single	669	33.8
Married	1189	60.1
Divorced/separated	105	5.3
Widowed	17	0.9
**Frequency Missing = 5**		
**Work (N = 1975)**		
Completely teleworking online from home	261	13.2
Completely working in the hospital	1005	50.9
Working from home and the hospital	683	34.6
Not working at all / on leave	26	1.3
**Frequency Missing = 10**		
**COVID-19 contact (N = 1962)**		
Yes	494	25.2
No	1468	74.8
**Frequency Missing = 23**		
**Healthcare Personnel (N = 1948)**		
Nurse	574	29.5
Physician	284	14.6
Allied Health Professional	578	29.7
Non-Clinical Staff	443	22.7
Researcher	69	3.5
**Frequency Missing = 37**		
**Hospital Department (N = 1965)**		
Clinical ^^^	1077	54.8
Non-Clinical	888	45.2
**Frequency Missing = 20**		
**Someone close affected by COVID-19 (N = 1956)+**		
None affected	1491	76.2
Quarantine	223	11.4
Infected	85	4.4
Hospitalized	111	5.7
Someone close to you died	46	2.4
**Frequency Missing = 29**		
Self-affected by COVID-19 (N = 1960)*		
None affected	1885	96.2
Quarantine	59	3.0
Infected	8	0.4
Hospitalized	8	0.4
**Frequency Missing = 25**		

The prevalence of mild-moderate psychological distress among HCWs in KSA was high; and severe distress was moderate Table 2. Combined, the incidence of at least mild psychological distress was almost 75%. Derived from the confidence interval, the overall prevalence of at least mild distress was 72.09%. Those who had directly engaged with the care of COVID-19 patients had high prevalence for both mild-moderate and severe psychological distress Table 2. The prevalence of mild psychological distress among those who had no contact with COVID-19 patients was also high Table 2.

**Table 2:** Prevalence of psychological distress (N = 1985).

Psychological Distress: K6 score	N	Prevalence		99% CI
		%	SE	
No/low distress	504	25.4	1.0	22–9–27.9
Mild/moderate	989	49.8	1.1	46–9–52.7
Severe	492	24.8	1.0	22–3–27.3
Mild/moderate/severe	1481	74.6	1.0	72–1–77.1
COVID-19 Contact*				
Yes	494			
No/low distress	99	20.0	1.8	15–4–24.7
Mild/moderate	230	46.6	2.2	40–8–52.4
Severe	165	33.4	2.1	27–9–38.9
Mild/moderate/severe	395	80.0	1.8	75–3–84.6
No	1468			
No/low distress	398	27.1	1.2	24–1–30.1
Mild/moderate	747	50.9	1.3	47–5–54.3
Severe	323	22.0	1.1	19–2–24.8
Mild/moderate/severe	1070	72.9	1.2	69–9–75.9

SE: Standard Error

*missing frequency = 23

The proportional odds model for non-modifiable correlates of psychological distress showed that females had increased odds of experiencing one level more of distress than males (OR = 1.23; 95% CI = 1.00–1.50) Table 3. The odds of increasing distress levels for those aged 40–49 and 50–59 decreased in comparison to those aged 20–29, with Ors ranging from 0.49–0.33. There was an 86% decrease in the odds of psychological distress for 60–70 year-olds vs. 20–29 year-olds (OR = 0.14; 95% CI = 0.07–0.27). Participants who reported working as researchers had 46% decrease in odds of elevated distress levels relative to other non-clinical staff (OR = 0.54; 95% CI = 0.33–0.90). Those who had engaged directly with COVID-19 patients were more likely to experience increasing distress than HCWs who did not have contact with infected patients (OR = 1.31; 95% CI = 1.05–1.63). It is clear that there were significantly increased odds of elevated distress levels for HCWs who had someone close to them undergo quarantine, hospitalization and/or death due to COVID-19 vs. those with closed loved ones unaffected by COVID-19. There were also increased odds of elevated distress for HCWs who had to quarantine themselves due to contracting COVID-19 vs. those who had not been infected by the disease.

**Table 3:** Proportional odds model with non-modifiable correlates predicting psychological distress (N = 1810).

Correlates	OR	95% CL		P-value
**Gender**				0.0*
Male	1.0	-	-	
Female	1.2	1.0	1.5	
**Age**				< .0001***
20–29	1.0	-	-	
30–39	0.8	0.6	1.1	
40–49	0.5	0.4	0.7	
50–59	0.3	0.2	0.5	
60–70	0.1	0.1	0.3	
**Marital Status**				0.8
Single	1.0	-	-	
Married	1.1	0.9	1.4	
Divorced/Separated	1.2	0.7	1.8	
Widowed	1.0	0.4	2.6	
**Healthcare Personnel**				0.0**
Nurse	1.1	0.8	1.5	
Physician	0.7	0.5	1.1	
Allied Health Professional	1.0	0.8	1.4	
Researcher	0.5	0.3	0.9	
Non-Clinical Staff	1.0	-	-	
**Hospital Department**				0.2
Clinical	1.2	0.9	1.4	
Non-Clinical	1.0	-	-	
**COVID-19 Contact**				0.0*
Yes	1.3	1.0	1.6	
No	1.0	-	-	
Someone close to you affected by COVID-19*				< .0001***
None affected	1.0	-	-	
Quarantine	1.5	1.1	2.0	
Infected	1.3	0.8	2.0	
Hospitalized	2.2	1.5	3.2	
Died	2.3	1.3	4.2	
**Self-affected by COVID-19^**				0.0**
None affected	1.0	-	-	
Quarantine	2.3	1.3	3.9	
Infected	2.6	0.6	11.5	
Hospitalized	1.8	0.5	7.0	

*p ≤ .05

**p ≤ .01

***p < .001

After controlling for non-modifiable correlates of psychological distress (reduced model; Table 4), the proportional odds model with modifiable correlates (Model A: financial impact, insomnia, work mode, and other worry-related factors) significantly predicted distress severity. Models with modifiable correlates added individually also showed a significant effect on psychological distress (Models B to R). However, when incremental differences (after controlling for non-modifiable correlates) were considered according to increased significance, the leading correlates of distress severity among HCWs in KSA were insomnia (Model B), worry about COVID-19 generally (Model C), feeling lonely, bored and missing being with friends (Model D), becoming infected by COVID-19 (Model E), the world not returning to what it was before the pandemic (Model F), continuation of the pandemic for a long time (Model G), not being able to practice favourite hobbies due to self-isolation (Model H), infecting close people (Model I), being far from family and loved ones because of the travel ban (Model J), and decline in fitness level and gaining weight due to self-isolation (Model K) Table 4.

**Table 4:** Likelihood ratios for proportional odds models with non-modifiable and modifiable correlates predicting psychological distress.

**Model**	**Predictors**	***N***	**χ** ^**2**^	**DF**	**Pr > ChiSq**
Reduced Model	Gender, age, marital status, COVID-19 contact, healthcare personnel, hospital department, someone close affected by COVID-19, self-affected by COVID-19	1810	194.76	21	< .0001
^†^Full Models			**Incremental χ** ^**2**^	**Incremental DF**	**Incremental Pr > ChiSq**
Model A	Financial impact, insomnia, work mode, all other worry-related factors	1671	863.6	41	< .0001
Model B	Insomnia	1806	506.7	2	< .0001
Model C	Worry about COVID-19 right now	1792	288.4	3	< .0001
Model D	Worry about feeling lonely, bored and miss being with friends	1794	264.5	2	< .0001
Model E	Worry about getting infected with COVID-19	1806	184.4	2	< .0001
Model F	Worry about the world not returning to what it was before the COVID-19 pandemic	1796	152.9	2	< .0001
Model G	Worry about continuation of the COVID-19 pandemic for a long time	1802	132.5	2	< .0001
Model H	Worry about not practicing favorite hobbies due to self- isolation	1800	113.5	2	< .0001
Model I	Worry about infecting close people with COVID-19	1806	112.7	2	< .0001
Model J	Worry about being far from family and loved ones because of the travel ban	1800	95.4	2	< .0001
Model K	Worry about decline in fitness level and gaining weight due to self-isolation	1801	89.5	2	< .0001
Model L	Worry about being stigmatized if infected by COVID-19	1795	87.9	2	< .0001
Model M	Worry about yourself, relatives or friends dying from infection with COVID-19	1797	83.2	2	< .0001
Model N	Worry about inability to obtain medical care or medications for you or your family	1797	56.4	2	< .0001
Model O	Worry about family and friends becoming infected with COVID-19	1799	54.8	2	< .0001
Model P	Financial impact	1802	49.2	3	< .0001
Model Q	Worry about unavailability of personal protective equipment (such as masks, gloves, gowns, and eyewear)	1802	35.7	2	< .0001
Model R	Worry about inability to obtain food and supplies needed for you or your family	1799	34.0	2	< .0001
Model S	Worry about losing your job and any financial resources	1797	12.2	2	< .0001
Model T	Work mode	1806	8.6	3	< .0001

^†^controlling for correlates in the reduced model

## Discussion

This study was one of the first in the Gulf Council Cooperation (GCC) region to conduct a cross-sectional survey on the prevalence and severity of psychological distress among HCWs in KSA during the COVID-19 pandemic. While several other studies have explored the psychological impact of COVID-19 on HCWs and the general public using different scales in KSA [39–41], our study looked at psychological distress in HCWs at the national level [39, 42], included a larger sample taken from six major hospitals, and examined psychological distress in general rather than focusing on specific disorders [40, 41, 43, 44]. Furthermore, while our results are in line with global findings, Saudi Arabia is culturally different in terms of its psychological landscape [45], and it is possible that religious and cultural beliefs impacted how HCWs responded to the survey. A study conducted in Qatar, a country considered relatively similar to Saudi Arabia in terms of culture and religion, found that Arab survey takers tend to be wary of the reliability and intentions of surveys, whereas for non-Arabs, their behavior towards surveys depends on their willingness as well as the survey’s perceived cognitive and time burden [46]. It has also been found that preference falsification is likely to be present in some Arab countries [47].

We found a high prevalence of mild-moderate and severe psychological distress among HCWs who had engaged in caring for COVID-19 patients, as well as those who were not involved. Gender, age, type of healthcare personnel, contact with COVID-19 patients, having someone close affected by COVID-19 and COVID-19 infection status of the HCW significantly predicted increased levels of psychological distress among the HCWs.

The prevalence of mild-moderate and severe psychological distress was high among HCWs in KSA, similar to the findings of other recent studies [2, 3, 6, 20]. Women were more likely than men to experience increasing psychological distress, consistent with other studies among HCWs [2, 3]. Those in the older age groups (40–70 years old vs. 20–29 years old) had decreased odds of experiencing higher distress. A similar trend was reported in a general population study [48], which posited that younger individuals interacting more with social media potentially encountered more triggers for distress. At the same time, several reports [3, 48, 49] emphasize that the elderly and older individuals still need to be considered with regards to mental health as they are more vulnerable to COVID-19 infection and are witnessing higher mortality rates within their age group.

Several studies conducted in neighboring countries between March and May 2020 also focused on psychological distress levels in HCWs. Authors of a study in Jordan reported depression in 21.2% and anxiety in 11.3% of HCWs; another study in Oman demonstrated moderate-to-severe anxiety in 26% of HCWs. Of note, the difference in figures could be a result of the use of different evaluation tools and the use of different classifications, even if the same scale was used [39].

With respect to type of healthcare personnel, in line with other studies [2, 50], nurses had increased odds (vs. nonclinical HCWs) of experiencing higher distress levels; however, this association was not significant. We found that nonclinical workers are significantly more distressed than researchers, consistent with a previous study [5] that indicated nonclinical workers were experiencing more distress than other types of HCWs during the pandemic. This could be because nonclinical HCWs are more representative of the general population. It has been reported that the distress of nonclinical HCWs is more severe than the distress of frontline workers caring for COVID-19 patients [9]. In terms of COVID-19 exposure, our findings also suggest that HCWs who had directly engaged in the diagnosis, treatment, or care of COVID-19 patients had increased odds of elevated distress levels compared to those who had not been in contact; this aligns with other reports [2, 3]. Given these results, healthcare organizations and hospitals must cater to the mental health needs of HCWs in contact with the infection, and develop specific interventions targeted at both medical and nonmedical staff (interventions will be discussed in further detail below).

Furthermore, those HCWs who had someone close to them in quarantine, hospitalized and/or who had died due to COVID-19, were more likely than those with unaffected loved ones to experience increased levels of distress. Additionally, HCWs who were under quarantine due to a COVID-19 diagnosis (vs. those unaffected) had increased odds of psychological distress, in line with previous studies [51]. These findings were consistent with evidence indicating that sources of distress among HCWs include concerns about health of loved ones, and health of self, wherein the perception of personal danger intensifies [2, 52]. Psychological interventions for HCWs ‒ affected by the pandemic ‒ must therefore ensure addressing risk factors such as potential harm to self and loved ones [17], and the psychological impact related to affected family members [15, 53].

In the multivariate analyses, adjusting for non-modifiable correlates of psychological distress, all modifiable correlates including financial impact, insomnia, work mode, and other worry-related factors remained significantly associated with psychological distress. However, by ranking incremental differences and significance, we found that the correlate most worth modifying among HCWs in KSA was insomnia. Other studies have also emphasized this risk factor and its impact in developing mental health problems including distress, especially during the pandemic [7, 20, 54]. Mental health treatment and intervention approaches would thus be more effective if they pro-actively incorporated plans to tackle sleep quality of HCWs, which can in turn alleviate their distress severity. A study conducted during the COVID-19 pandemic suggests implementing a customized shift system catered to health care workers in high-pressured roles as one possible intervention [55].

Worry about COVID-19 generally, loneliness, boredom and separation from friends, getting infected, infecting close people, and being far from family and loved ones because of the travel ban were other significant correlates that predicted increasing distress levels in the adjusted multivariate analyses. Recent reports affirm that loneliness, boredom, separation from loved ones and uncertainty over disease status can have a dramatic psychological impact during a pandemic [51, 53, 56]. As the transmission of COVID-19 can occur through asymptomatic carriers, psychological distress levels may exacerbate if an individual fears themselves to be the carrier [8]. Concerns about infecting family members (especially those most at risk to contract the infection); [3, 12], and contracting the infection themselves as the number of suspected and confirmed cases continues to increase, contribute to the pressures that the HCWs undergo daily [2, 57]. For these reasons, HCWs, along with the elderly and people suffering from autoimmune diseases, were given priority by the Saudi Ministry of Health when vaccinations and treatments became available [58, 59]. Moreover, with personal travel being curtailed and travel restrictions in place (locally and globally) to limit the spread of infection [58], separation from family results in psychological issues [17, 60]. Du et al. [6] emphasize that strong family support ‒ which due to separation may fall short of being adequate ‒ is vital in increasing frontline HCWs’ resilience to stress during a public health emergency. Various initiatives were launched to support HCWs, including ‘Da’em’, a program launched by the Saudi Commission for Health Specialties to support health practitioners through specialists and various support programs during the pandemic. In addition, King Faisal Specialist Hospital & Research Centre also provided a mental health and psychosocial support hotline in Arabic [53, 61].

Finally, we found that worry about the world not returning to what it was before the pandemic, continuation of the pandemic for a long time, concerns about not engaging with hobbies, and decline in fitness level and weight gain due to self-isolation were some of the other correlates that significantly predicted psychological distress among HCWs in KSA. A similar report from Italy also found that locals experienced high levels of stress due to not having an estimate of how long the pandemic would last and how long their lives would continue to be disrupted [10]. Indeed, collective anxiety and growing fear among communities are the consequence of mass quarantine [62]. In a Chinese study, individuals who exercised regularly reported feeling less satisfied; thus, COVID-19 mental health interventions may need to consider the needs of more physically active individuals, who might experience more distress due to the restrictions posed by the outbreak [21].

Our findings underline the need for psychological intervention plans to address the distress experienced by HCWs in KSA, and are similar to global findings [7, 12, 63]. In addition, our findings can help nationwide strategic decision-makers and policy makers consider the outlined psychological risk factors and prepare for major disasters like the COVID-19 by developing and launching accessible, cost-effective mental health services and recovery programs for HCWs [7, 48]. Telehealth and e-technology services offering virtual peer support, counseling, online resources (e.g., psycho-education), mobile apps, psychological assistance hotlines have been reported to be beneficial in tackling specific sources of distress among HCWs [3, 5, 16, 64, 65]. Previous reports also highlight support provided by healthcare management and direct managers; for example, disseminating clear communications about the disease and logistics related to their work [1, 6, 51, 53] and strengthening personnel via trainings to provide a timely mental health response during emergencies such as the COVID-19 pandemic [17].

This study has some limitations and findings must be interpreted in their light. First, the survey was self-reported and completed by HCWs at a single time point. Longitudinal studies using multiple standardized measures are needed to assess the psychological impact of the pandemic among HCWs. As HCWs may face major changes on a daily basis, monitoring their mental health outcomes over time and prioritizing them is crucial to ensure the quality of care provided to patients [1, 3]. Second, our findings are limited to selected regions of the KSA and cannot be generalized to other differently affected national regions. Third, data about HCWs’ work hours and workload might have provided more detail about the associated distress [6]. Finally, although face validity was carried out to some extent, given the urgency of the topic and shortage of time, most of the questionnaire items did not undergo psychometric testing.

## Conclusion

This cross-sectional study found that the prevalence of psychological distress reported by HCWs in KSA was high, ranging in severity from mild-moderate to severe. Younger HCWs, women, those in contact with COVID-19 patients, those with loved ones affected by COVID-19, and those personally affected by COVID-19 were the most at risk of experiencing elevated distress levels. To effectively address psychological distress among HCWs, public health policy makers and mental health professionals must give special attention to risk factors such as insomnia, loneliness, fear of transmission, and separation from loved ones.

## Supporting information

S1 Fig Key events during the outbreak of COVID-19 in KSA and study timeline. (TIF)

S1 Table Details and distribution of healthcare personnel. (DOCX)

S2 Table Variable means. (DOCX)

S3 Table Variable frequencies. (DOCX)

S4 Table Score test for the proportional odds assumption. (DOCX)

S5 Table Binary regression model of non-modifiable correlates with stigma predicting psychological distress. (DOCX)

## Abbreviations

GCC: Gulf Council Cooperation
HCWs: Healthcare workers
KSA: Kingdom of Saudi Arabia
KFSH&RC: King Faisal Specialist Hospital & Research Centre
SNMHS: Saudi National Mental Health Survey
