# Supplementary figures and images for: Psychological distress reported by healthcare workers in Saudi Arabia during the COVID-19 pandemic: A cross-sectional study
Source: PLoS One. 2022 Jun 3;17(6):e0268976. doi: 10.1371/journal.pone.0268976 (PMC9165802; doi:10.1371/journal.pone.0268976)

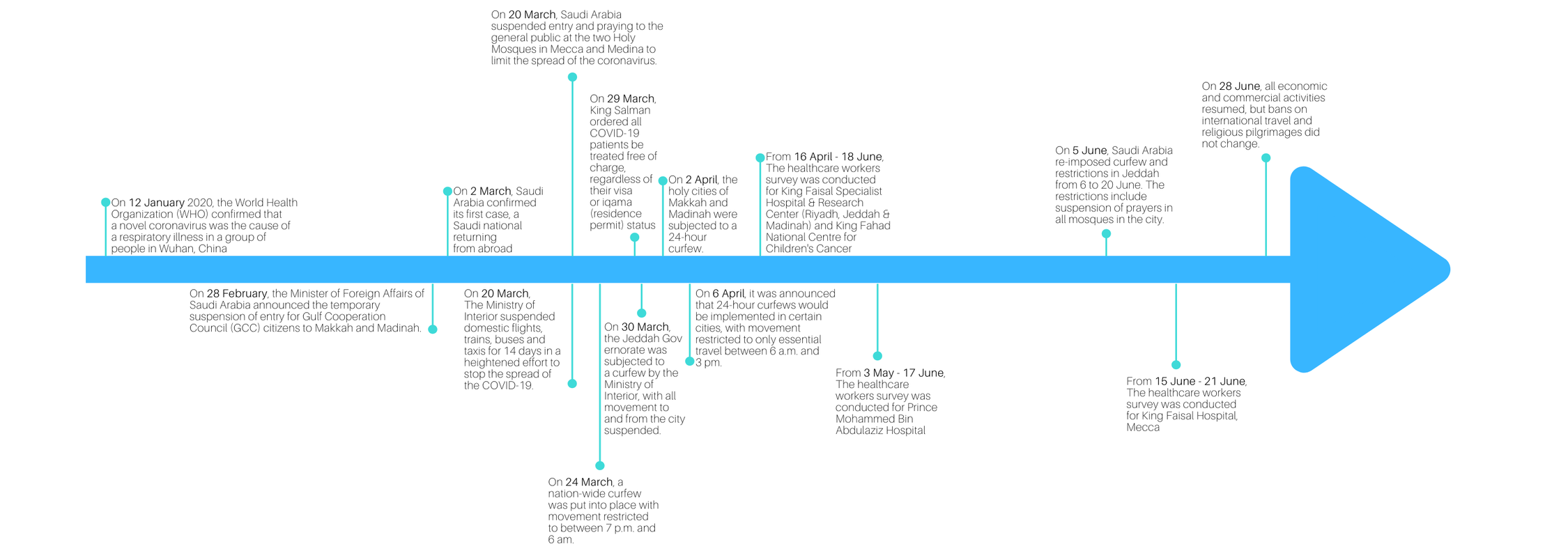

Supplement: S1 Fig — (TIF) [file pone.0268976.s001.tif]
