# Supplementary material for: Psychological distress reported by healthcare workers in Saudi Arabia during the COVID-19 pandemic: A cross-sectional study
Source: PLoS One. 2022 Jun 3;17(6):e0268976. doi: 10.1371/journal.pone.0268976 (PMC9165802; doi:10.1371/journal.pone.0268976)
Supplement: S1 Table — (DOCX) [file pone.0268976.s002.docx]

**S1 Table. Details and distribution of Healthcare personnel**

(i) Details of Healthcare personnel categories:

| **Nurse** includes: Nurse, Head Nurse, Nurse Educator, Clinical Care Coordinator |
| --- |
| **Physician** includes: Physicians, Doctors, Consultants |
| **Allied Health Professionals** includes: Allied Health Sciences, Audiologist, Care Assistant, Case Manager, Clinical Dietitian, Dental Assistant, Dietician, Dietician Assistant, Health Education Specialist, Infection Control Coordinator, Infection Control Practitioner, Medical Physicist, Medical Transcriptionist, Nursing Care Assistant, Optometrist, Orthotist and Prosthetist, Paramedic, Patient Care Assistant, Patient Relation rep., Perioperative, Physical Therapist, Radiation Oncology Physicist, Radiology Support Service Representative, Social Services, Social Worker, Therapeutic Medical Physicist, Transplant Coordinator |
| **Nonclinical Staff** includes: Administrative Assistant, Administrative Coordinator, Administrative Staff, Audiovisual Technician, Bed Utilization, Diet Clerk, Health Informatics, Healthcare Quality Professional, Healthcare Information Technology Affairs, Hospital Assistant, Housing Supervisor, HR Administration, Insurance department, Interpreter, Integration Analyst, Medical Coder, Medical Store, Audiovisual Department, Patients flow, Product Coordinator, Program Director, Quality Management Department, Quality Coordinator, Quality Professional, Safety Officer, Career Development Program, Supply Chain Management, Secretary, Security, Central Patient Transport, Shop Planning Assistant, Utilities Department, Utility and maintenance, Ward Clerk, Civil Works, Clinical Engineer, CSSD technician, Engineer, Engineering Project Management, Engineering/Maintenance, Housekeeping Supervisor, Maintenance Dept., Plant Operator, Senior Clinical Engineer, Supervisor Environmental Service, Support Services, Technical/support, Technician, Water Treatment Operator |
| **Researcher** includes: Clinical Analyst, Clinical Research Coordinator, Clinical Research Coordinator (Trainee), Research Coordinator, Senior SAS Programmer/Analyst, Technical Specialist, Trainee at Research Centre |

(ii) Distribution by department (N = 1985)

| **Department** | **n** |
| --- | --- |
| Clinical: |  |
| Anesthesiology | 32 |
| Critical care medicine | 100 |
| Dentistry | 28 |
| Dermatology | 5 |
| Emergency medicine | 63 |
| Family medicine/polyclinics | 36 |
| Heart centre | 76 |
| Medical genetics | 11 |
| ^a^Medicine | 129 |
| Mental health | 6 |
| Neurosciences | 31 |
| Obstetrics & gynecology | 26 |
| Oncology centre | 97 |
| Radiation therapy | 13 |
| Ophthalmology | 8 |
| Orthopedic surgery | 22 |
| Orthotics/prosthetics services | 4 |
| Otolaryngology/ head & neck surgery/ & communication sciences | 21 |
| Pathology & laboratory medicine | 97 |
| Pediatric hematology/oncology | 23 |
| Pediatrics | 66 |
| Radiology | 76 |
| Surgery | 99 |
| Urology | 8 |
| ^b^Non clinical:  Other | 888 |
| Missing data | 20 |

**^a^Department of Medicine** consists of subspecialty units (Allergy/Immunology, Endocrinology, Gastroenterology, Infectious Diseases, Internal Medicine, Nephrology, Pulmonary Medicine, and Rheumatology), and medical units (Sleep Medicine Unit, Hemodialysis Unit, and Endoscopy).

**^b^Nonclinical departments** include: Human Resources, Pharmacy, Nutrition, Social services, Mortuary, Administration, Research, Medical Error, Patient Experience, Medical records, Health insurance, Public Health, Infection Control, Information Technology, Legal Affairs, Health Education, and other non-specified hospital offices, units and wards.
