# Supplementary material for: Psychological distress reported by healthcare workers in Saudi Arabia during the COVID-19 pandemic: A cross-sectional study
Source: PLoS One. 2022 Jun 3;17(6):e0268976. doi: 10.1371/journal.pone.0268976 (PMC9165802; doi:10.1371/journal.pone.0268976)
Supplement: S2 Table — (DOCX) [file pone.0268976.s003.docx]

| **S2 Table. Variable means** | | | | | |
| --- | --- | --- | --- | --- | --- |
| **Variables** | **N** | **Mean** | **Std Dev** | **Minimum** | **Maximum** |
| **Age** | 1927 | 38.7 | 9.8 | 20 | 70 |
| **K6 Scores** | 1985 | 8.9 | 5.6 | 0 | 24 |
