# Supplementary material for: Psychological distress reported by healthcare workers in Saudi Arabia during the COVID-19 pandemic: A cross-sectional study
Source: PLoS One. 2022 Jun 3;17(6):e0268976. doi: 10.1371/journal.pone.0268976 (PMC9165802; doi:10.1371/journal.pone.0268976)
Supplement: S3 Table — (DOCX) [file pone.0268976.s004.docx]

| **S3 Table. Variable Frequencies** |  |  |
| --- | --- | --- |
| **Socio-demographic Variables** | **N** | **%** |
| **K6 Scores (N=1985)** |  |  |
| No/low distress | 504 | 25.4 |
| Mild/moderate | 989 | 49.8 |
| Severe | 492 | 24.8 |
| **K6 Scores (N=1985)** |  |  |
| Mild/moderate/severe | 1481 | 74.6 |
| No/low distress | 504 | 25.4 |
| **Gender (N=1978)** |  |  |
| Male | 756 | 38.2 |
| Female | 1222 | 61.8 |
| Frequency Missing = 7 |  |  |
| **Age (N=1927)** |  |  |
| 20-29 | 360 | 18.7 |
| 30-39 | 765 | 39.7 |
| 40-49 | 479 | 24.9 |
| 50-59 | 270 | 14.0 |
| 60-70 | 53 | 2.8 |
| Frequency Missing = 58 |  |  |
| **Marital Status (N=1980)** |  |  |
| Single | 669 | 33.8 |
| Married | 1189 | 60.1 |
| Divorced/separated | 105 | 5.3 |
| Widowed | 17 | 0.9 |
| Frequency Missing = 5 |  |  |
| **Work (N=1975)** |  |  |
| Completely teleworking online from home | 261 | 13.2 |
| Completely working in the hospital | 1005 | 50.9 |
| Working from home and the hospital | 683 | 34.6 |
| Not working at all / on leave | 26 | 1.3 |
| Frequency Missing = 10 |  |  |
| **COVID-19 contact (N=1962)** |  |  |
| Yes | 494 | 25.2 |
| No | 1468 | 74.8 |
| Frequency Missing = 23 |  |  |
| **Healthcare Personnel (N=1948)** |  |  |
| Nurse | 574 | 29.5 |
| Physician | 284 | 14.6 |
| Allied Health Professional | 578 | 29.7 |
| Non-Clinical Staff | 443 | 22.7 |
| Researcher | 69 | 3.5 |
| Frequency Missing = 37 |  |  |
| **Hospital Department (N=1965)** |  |  |
| Clinical ^^^ | 1077 | 54.8 |
| Non-Clinical | 888 | 45.2 |
| Frequency Missing = 20 |  |  |
| **Someone close affected by COVID-19 (N=1956)** |  |  |
| None affected | 1491 | 76.2 |
| Quarantine | 223 | 11.4 |
| Infected | 85 | 4.4 |
| Hospitalized | 111 | 5.7 |
| Someone close to you died | 46 | 2.4 |
| Frequency Missing = 29 |  |  |
| **Self-affected by COVID-19 (N=1960)** |  |  |
| None affected | 1885 | 96.2 |
| Quarantine | 59 | 3.0 |
| Infected | 8 | 0.4 |
| Hospitalized | 8 | 0.4 |
| Frequency Missing = 25 |  |  |
| **Financial Impact (N=1957)** |  |  |
| Not applicable | 1213 | 62.0 |
| You or another jobholder in your household had an increase in work hours | 230 | 11.8 |
| You experienced another loss of income (retirement payments, stocks, other investments) | 200 | 10.2 |
| You or another jobholder in your household lost a job, were laid off, or had hours reduced | 314 | 16.0 |
| Frequency Missing = 28 |  |  |
| **Insomnia (N=1978)** |  |  |
| Always | 481 | 24.3 |
| Sometimes | 1083 | 54.8 |
| Never | 414 | 20.9 |
| Frequency Missing = 7 |  |  |
| **Worry about COVID-19 right now (N=1959)** |  |  |
| Not worried at all | 87 | 4.4 |
| Not very worried | 311 | 15.9 |
| Somewhat worried | 868 | 44.3 |
| Very worried | 693 | 35.4 |
| Frequency Missing = 26 |  |  |
| **Worry about getting infected with COVID-19 (N=1976)** |  |  |
| Does not worry me at all | 250 | 12.7 |
| Worries me somewhat | 1013 | 51.3 |
| Worries me a lot | 713 | 36.1 |
| Frequency Missing = 9 |  |  |
| **Worry about infecting close people with COVID-19 (N=1976)** |  |  |
| Does not worry me at all | 157 | 8.0 |
| Worries me somewhat | 549 | 27.8 |
| Worries me a lot | 1270 | 64.3 |
| Frequency Missing = 9 |  |  |
| **Worry about unavailability of personal protective equipment (such as masks, gloves, gowns, and eyewear) (N=1970)** |  |  |
| Does not worry me at all | 479 | 24.3 |
| Worries me somewhat | 745 | 37.8 |
| Worries me a lot | 746 | 37.9 |
| Frequency Missing = 15 |  |  |
| **Worry about family and friends becoming infected with COVID-19 (N=1970)** |  |  |
| Does not worry me at all | 106 | 5.4 |
| Worries me somewhat | 459 | 23.3 |
| Worries me a lot | 1405 | 71.3 |
| Frequency Missing = 15 |  |  |
| **Worry about yourself, relatives or friends dying from infection with COVID-19 (N=1963)** |  |  |
| Does not worry me at all | 206 | 10.5 |
| Worries me somewhat | 568 | 28.9 |
| Worries me a lot | 1189 | 60.6 |
| Frequency Missing = 22 |  |  |
| **Worry about feeling lonely, bored and miss being with friends (N=1963)** |  |  |
| Does not worry me at all | 384 | 19.6 |
| Worries me somewhat | 915 | 46.6 |
| Worries me a lot | 664 | 33.8 |
| Frequency Missing = 22 |  |  |
| **Worry about being far from family and loved ones because of the travel ban (N=1966)** |  |  |
| Does not worry me at all | 298 | 15.2 |
| Worries me somewhat | 637 | 32.4 |
| Worries me a lot | 1031 | 52.4 |
| Frequency Missing = 19 |  |  |
| **Worry about losing your job and any financial resources (N=1967)** |  |  |
| Does not worry me at all | 609 | 31.0 |
| Worries me somewhat | 633 | 32.2 |
| Worries me a lot | 725 | 36.9 |
| Frequency Missing = 18 |  |  |
| **Worry about inability to obtain food and supplies needed for you or your family (N=1969)** |  |  |
| Does not worry me at all | 646 | 32.8 |
| Worries me somewhat | 725 | 36.8 |
| Worries me a lot | 598 | 30.4 |
| Frequency Missing = 16 |  |  |
| **Worry about inability to obtain medical care or medications for you or your family (N=1966)** |  |  |
| Does not worry me at all | 516 | 26.3 |
| Worries me somewhat | 698 | 35.5 |
| Worries me a lot | 752 | 38.3 |
| Frequency Missing = 19 |  |  |
| **Worry about continuation of the COVID-19 pandemic for a long time (N=1971)** |  |  |
| Does not worry me at all | 75 | 3.8 |
| Worries me somewhat | 512 | 26.0 |
| Worries me a lot | 1384 | 70.2 |
| Frequency Missing = 14 |  |  |
| **Worry about decline in fitness level and gaining weight due to self-isolation (N=1968)** |  |  |
| Does not worry me at all | 450 | 22.9 |
| Worries me somewhat | 809 | 41.1 |
| Worries me a lot | 709 | 36.0 |
| Frequency Missing = 17 |  |  |
| **Worry about not practicing favorite hobbies due to self- isolation (N=1967)** |  |  |
| Does not worry me at all | 558 | 28.4 |
| Worries me somewhat | 870 | 44.2 |
| Worries me a lot | 539 | 27.4 |
| Frequency Missing = 18 |  |  |
| **Worry about the world not returning to what it was before the COVID-19 pandemic (N=1966)** |  |  |
| Does not worry me at all | 312 | 15.9 |
| Worries me somewhat | 798 | 40.6 |
| Worries me a lot | 856 | 43.5 |
| Frequency Missing = 19 |  |  |
| **Worry about being stigmatized if infected by COVID-19 (N=1963)** |  |  |
| Does not worry me at all | 630 | 32.1 |
| Worries me somewhat | 664 | 33.8 |
| Worries me a lot | 669 | 34.1 |
| Frequency Missing = 22 |  |  |
