# Supplementary material for: Psychological distress reported by healthcare workers in Saudi Arabia during the COVID-19 pandemic: A cross-sectional study
Source: PLoS One. 2022 Jun 3;17(6):e0268976. doi: 10.1371/journal.pone.0268976 (PMC9165802; doi:10.1371/journal.pone.0268976)
Supplement: S4 Table — (DOCX) [file pone.0268976.s005.docx]

| **S4 Table. Score test for the proportional odds assumption** | | | |
| --- | --- | --- | --- |
|  | **χ^2^** | **DF** | **Pr > ChiSq** |
| **Table 3** | 22.8 | 21 | 0.3545 |
| **Table 4 - Reduced Model** | 22.8 | 21 | 0.3545 |
| **Table 4 - Model A** | 78.3 | 60 | 0.0565 |
| **Table 4 - Model B** | 23.2 | 23 | 0.4477 |
| **Table 4 - Model C** | 26.4 | 24 | 0.3320 |
| **Table 4 - Model D** | 23.8 | 23 | 0.4124 |
| **Table 4 - Model E** | 34.6 | 23 | 0.0571 |
| **Table 4 - Model F** | 28.4 | 23 | 0.2016 |
| **Table 4 - Model G** | 32.5 | 23 | 0.0901 |
| **Table 4 - Model H** | 29.5 | 23 | 0.1647 |
| **Table 4 - Model I** | 30.8 | 23 | 0.1272 |
| **Table 4 - Model J** | 24.2 | 23 | 0.3926 |
| **Table 4 - Model K** | 30.2 | 23 | 0.1430 |
| **Table 4 - Model M** | 23.1 | 23 | 0.4555 |
| **Table 4 - Model N** | 25.3 | 23 | 0.3354 |
| **Table 4 - Model O** | 26.0 | 23 | 0.3024 |
| **Table 4 - Model P** | 29.7 | 24 | 0.1961 |
| **Table 4 - Model Q** | 26.9 | 23 | 0.2592 |
| **Table 4 - Model R** | 27.7 | 23 | 0.2271 |
| **Table 4 - Model S** | 27.9 | 23 | 0.2185 |
| **Table 4 - Model T** | 23.7 | 24 | 0.4813 |
