# Supplementary material for: Psychological distress reported by healthcare workers in Saudi Arabia during the COVID-19 pandemic: A cross-sectional study
Source: PLoS One. 2022 Jun 3;17(6):e0268976. doi: 10.1371/journal.pone.0268976 (PMC9165802; doi:10.1371/journal.pone.0268976)
Supplement: S5 Table — (DOCX) [file pone.0268976.s006.docx]

| **S5 Table. Binary regression model of non-modifiable correlates with Stigma predicting psychological distress.** | | | | | | | |
| --- | --- | --- | --- | --- | --- | --- | --- |
| **Non-modifiable correlates with Stigma** |  | **Mild/Severe Stress Vs. No Stress** | | | | | |
|  |  | **OR** |  | **95% CL** | |  | **P-value** |
| **Gender** |  |  |  |  |  |  | 0.1 |
| Male |  | 1.0 |  | - | - |  |  |
| Female |  | 1.2 |  | 0.9 | 1.6 |  |  |
| **Age** |  |  |  |  |  |  | <.0001*** |
| 20-29 |  | 1.0 |  | - | - |  |  |
| 30-39 |  | 0.8 |  | 0.6 | 1.2 |  |  |
| 40-49 |  | 0.6 |  | 0.4 | 0.9 |  |  |
| 50-59 |  | 0.4 |  | 0.2 | 0.6 |  |  |
| 60-70 |  | 0.1 |  | 0.1 | 0.3 |  |  |
| **Marital Status** |  |  |  |  |  |  | 0.4 |
| Single |  | 1.0 |  | - | - |  |  |
| Married |  | 1.1 |  | 0.8 | 1.5 |  |  |
| Divorced/Separated |  | 1.4 |  | 0.8 | 2.4 |  |  |
| Widowed |  | 0.6 |  | 0.2 | 1.7 |  |  |
| **Healthcare Personnel** |  |  |  |  |  |  | 0.4 |
| Nurse |  | 1.1 |  | 0.7 | 1.6 |  |  |
| Physician |  | 0.9 |  | 0.6 | 1.5 |  |  |
| Allied Health Professional |  | 1.0 |  | 0.7 | 1.4 |  |  |
| Researcher |  | 0.6 |  | 0.3 | 1.0 |  |  |
| Non-Clinical Staff |  | 1.0 |  | - | - |  |  |
| **Hospital Department** |  |  |  |  |  |  | 0.1 |
| Clinical |  | 1.2 |  | 0.9 | 1.7 |  |  |
| Non-Clinical |  | 1.0 |  | - | - |  |  |
| **COVID-19 Contact** |  |  |  |  |  |  | 0.5 |
| Yes |  | 1.3 |  | 1.0 | 1.6 |  |  |
| No |  | 1.0 |  | - | - |  |  |
| **Someone close to you affected by COVID-19** |  |  |  |  |  |  | 0.0*** |
| None affected |  | 1.0 |  | - | - |  |  |
| Quarantine |  | 2.1 |  | 1.3 | 3.2 |  |  |
| Infected |  | 1.4 |  | 0.8 | 2.6 |  |  |
| Hospitalized |  | 2.2 |  | 1.2 | 3.9 |  |  |
| Died |  | 2.1 |  | 0.9 | 5.1 |  |  |
| **Self-affected by COVID-19** |  |  |  |  |  |  | 0.0** |
| None affected |  | 1.0 |  | - | - |  |  |
| Quarantine/Infected/Hospitalized |  | 4.2 |  | 1.5 | 11.8 |  |  |
| **Worry about being stigmatized if infected by COVID-19^^^** | | |  |  |  |  | <.0001*** |
| Does not worry me at all |  | 0.3 |  | 0.2 | 0.4 |  |  |
| Worries me somewhat |  | 0.5 |  | 0.3 | 0.6 |  |  |
| Worries me a lot |  | 1.0 |  | - | - |  |  |
| *p ≤ .05; **p ≤ .01; ***p <.001 | | | | | | | |
| ^^^ The Quarantine, Infected and Hospitalized categories were combined due to low frequencies | | | | | | | |
